# Rabbit hemorrhagic disease virus VP60 protein expressed in recombinant swinepox virus self-assembles into virus-like particles with strong immunogenicity in rabbits

**DOI:** 10.3389/fmicb.2022.960374

**Published:** 2022-08-04

**Authors:** Changjin Liu, Min Lin, Huanyi Hu, Xiaolan Liu, Yanchao Bian, Xiaohua Huang, Xiaoxiang Li, Wenyang Yu, Feng Luo, Shunzhou Deng

**Affiliations:** ^1^Department of Preventive Veterinary Medicine, College of Animal Science and Technology, Jiangxi Agricultural University, Nanchang, Jiangxi, China; ^2^Jiangxi Jinyibo Biotechnology Company, Nanchang, Jiangxi, China

**Keywords:** rabbit hemorrhagic disease virus, VP60 protein, recombinant swinepox virus, virus-like particles, vaccine

## Abstract

Rabbit Hemorrhagic Disease (RHD) is an economically significant infectious disease of rabbits, and its infection causes severe losses in the meat and fur industry. RHD Virus (RHDV) is difficult to proliferate in cell lines *in vitro*, which has greatly impeded the progress of investigating its replication mechanism and production of inactivated virus vaccines. RHDV VP60 protein is a major antigen for developing RHD subunit vaccines. Herein, we constructed a TK-deactivated recombinant Swinepox virus (rSWPV) expressing VP60 protein and VP60 protein coupled with His-tag respectively, and the expression of foreign proteins was confirmed using immunofluorescence assay and western blotting. Transmission electron microscopy showed that the recombinant VP60, with or without His-tag, self-assembled into virus-like particles (VLPs). Its efficacy was evaluated by comparison with available commercial vaccines in rabbits. ELISA and HI titer assays showed that high levels of neutralizing antibodies were induced at the first week after immunization with the recombinant strain and were maintained during the ongoing monitoring for the following 13 weeks. Challenge experiments showed that a single immunization with 10^6^ PFU of the recombinant strain protected rabbits from lethal RHDV infection, and no histopathological changes or antigenic staining was found in the vaccine and rSWPV groups. These results suggest that rSWPV expressing RHDV VP60 could be an efficient candidate vaccine against RHDV in rabbits.

## Introduction

Rabbit hemorrhagic disease virus (RHDV), the causative agent of rabbit hemorrhagic disease (RHD), causes acute necrotizing hepatitis and disseminated intravascular coagulation with high fatality rates in adult European rabbits (Oryctolagus cuniculus; Abrantes et al., 2012). The disease was first identified in China in 1984 and since then RHDV has spread rapidly across the continent and within a few years led to outbreaks in most parts of the world. RHD causes significant economic losses to the rabbit meat and fur industry and has a significant negative ecological impact on hare populations and indirectly on the predators on which they depend.

RHDV, a member of the Lagoviruses genus in the Caliciviridae family, is a non-enveloped icosahedral virus possessing a single-stranded positive-sense RNA genome of approximately 7.4 kb in length, containing two slightly overlapping open reading frames (ORFs): ORF1 and ORF2. ORF1 encodes a polyprotein cleaved by the viral protease into seven nonstructural proteins (NSP1-7) and a major structural capsid protein VP60 at its C-terminus (Abrantes et al., 2012). The capsid virus protein (VP60), which can self-assemble to form virus-like particles (VLPs) of approximately 33–40 nm, is a major target of host immune defense against RHDV and plays an important role in virus diagnosis and vaccine design (Kong et al., 2015).

Disease control in rabbits relies heavily on vaccination and biosecurity measures. Commercially available vaccines are still traditionally inactivated viruses collected from artificially infected rabbit organs. Due to the difficulty of growing RHDV in cell culture, the current vaccines are unacceptable from both safety and ethical perspectives (Zhu et al., 2017). Thus, the cloning and expression of major immunogenic proteins in protein expression systems provide avenues for RHDV vaccine research. VLPs self-assemble upon expression of the RHDV capsid virus protein (VP60) and are an effective vaccine that provides complete protection for extended periods (Fernandez et al., 2011; Song et al., 2017; Muller et al., 2019). RHDV VLPs are structurally and immunologically identical to natural viral particles in a variety of protein expression recombinant vectors, including baculovirus, Lactobacillus casei, adenovirus, Escherichia coli, Orf virus, Pichia pastoris, and myxoma virus (Barcena et al., 2000; Farnos et al., 2006, 2009; Fernandez et al., 2011; Rohde et al., 2011; Guo et al., 2016; Jiang et al., 2018; Wang et al., 2019; Qi et al., 2020).

Poxviruses have been used as vectors for human and veterinary vaccines (Bhanuprakash et al., 2012; García-Arriaza and Esteban, 2014). Swinepox virus (SWPV) belongs to the genus Suipoxvirus of the family Poxviridae and has biological properties, such as host specificity, mild pathogenicity, good thermal stability, and low transmission rate. As with other poxviruses of livestock, SWPV has been developed as a recombinant vaccine vector for the expression of antigens from swine pathogens, such as Porcine circovirus type 2 (PCV2; Lin et al., 2012), swine fever virus (CSFV; Hahn et al., 2001), swine influenza virus (SIV; Xu et al., 2012), transmissible gastroenteritis virus (TGEV; Yuan et al., 2015), porcine reproductive and respiratory syndrome virus (PRRSV), and porcine epidemic diarrhea virus (PEDV), and it has been shown to be effective. SWPV demonstrating strict host specificity has been used to develop as a non-productively replicating viral vector for use in non-pigs, mammalian species, such as the cat (Winslow et al., 2003). The biosafety assessment of SWPV vector-based vaccines showed that SWPV is not infectious at 60 h after vaccination in a rabbit model.

This study aimed to expand alternative vectors for RHDV VP60 protein expression, and we describe the construction of a recombinant SWPV expressing RHDV VP60 protein and evaluate its potential to protect rabbits from RHDV infection.

## Materials and methods

### Cells, viruses, and reagents

PK15 cells and IPEC-J2 cells were grown in Dulbecco’s Modified Eagle’s medium (DMEM; Invitrogen, United States) containing 5% (vol/vol) heat-inactivated fetal bovine serum (FBS), 100 unit/ml of penicillin/streptomycin (Solarbio, China) and 2 mM L-glutamine (Sigma-Aldrich, United States). Cell cultures were maintained at 37°C in a humidified incubator with 5% CO_2_. Wild-type swinepox virus (strain SWPV-JX20G, GenBank accession no. OL456209) was isolated from the skin scabs of pigs in our laboratory. The VP60 gene of the classic RHDV (GenBank accession no. KP144791.2) was synthesized and Sus scrofa codon-optimized by GenScript Biological Technology Co., Ltd. (Nanjing, China) and cloned into the pUC19 plasmid. Mouse monoclonal antibody (mAb) 4D5 directed against VP60 is made and stored in our laboratory. Mouse antibody against cellular GAPDH (Glyceraldehyde-3-phosphate dehydrogenase) was purchased from Proteintech Biological Technology Co., Ltd. (Wuhan, China).

### Construction of plasmid transfer vector pSWE11-28V and pSWE11-28VH

The procedure used to construct the recombinant SWPV virus was adapted from the previously described standard method (Fan and Lin, 2016). The poxviruses TK locus has been traditionally used for insertion of heterologous sequences, so the TK deletion transfer vector was constructed based on pUC19 plasmid. Primers TKL-F and TKL-R containing EcoR I and Xba I restriction sites were used to amplify a 1,026-bp fragment including the partial TK gene as the left arm of homologous recombination with the genomic viral DNA as a template. This fragment was inserted into pUC19 previously digested with the same restriction enzymes. The right arm (1,058-bp) was also amplified using Primers TKR-F and TKR-R digested with Xba I and Hind III to construct the pSW plasmid. A cassette containing the enhanced green fluorescent protein (EGFP) under the control of the vaccinia virus P11 promoter (Senkevich et al., 2004) was cloned into the Xba I-Not I site of pSW and introduced a Sma I restriction site to yield the pSWE11 plasmid. The resulting plasmid (pSWE11) was subsequently digested with Sma I to insert VP60 or VP60-his fragment (generated by primers P28VP60-F, P28VP60-R, and P28VP60-His-R, both fragments under the control of the modified promoter P28 sequence; Davison and Moss, 1989) used the In-Fusion cloning technique (Zhu et al., 2007) to construct the pSWE11-28 V or pSWE11-28VH. Correct Insertion of those genes was verified by restriction enzyme analysis and DNA sequencing. The primers sequences for construction are shown in Table 1.

**Table 1:** Oligonucleotide sequences of the primers used for cloning and detecting.

Primer	Oligonucleotide sequences(5′-3′)
TKL-F	CGGAATTCCACGTTGAAACACTCGTTACTGA
TKL-R	CGTCTAGATGTCCTTCGTCTA TTCCAACAAT
TKR-F	CGTCTAGAGCGGCCGCCTCGAGCGCGGGTACCTAGTGATAAATTCTTTAATGATATTGT
TKR-R	CGAAGCTTTTGACCCTGATATGGAAACTTTTTA
P28VP60-F	TCTCAAAGTCTGCCCCTTTTTTTTTTTTTTTTTTTTGGCATATAAATGGAGGGCAAAGCCCGCAC
P28VP60-R	CCTCGAGCCGCGGTTAGACATAAGAAAAGCCA
P28VP60-His-R	CCTCGAGCCGCGGTTAATGATGGTGGTGATGATGGACATAAGAAAAGCCA
TK309-F	GGACCTATGTTCTCTGGAAAGAGT
TK309-R	TGTTCCATCTAATGCAGCAACG
SWPV-qPCR-F SWPV-qPCR-R SWPV-qPCR-P	CTGAGATTGCGACGTAACAGAGTT TGCCACTGGTGATATAACCGAAT Fam-TGTCTCTTTTAGTGCGTTATTA-Tamra

### Generation and identification of recombinant SWPVs

The recombinant SWPVs were isolated by the homologous recombination approach by adaptation of previous procedures (Fan and Lin, 2016). Cultured PK15 cells (1 × 10^6^ cells per well in a 6-well plate) were infected with parental SWPV-JX20G virus at a multiplicity of infection (MOI) of 1 PFU/cell and transfected 4 h later with 4 μg of DNA plasmid pSWE11-28 V and pSWE11-28VH, using Lipofectamine 2000 reagent according to the manufacturer’s recommendations (Invitrogen, United States). At 96 hpi, the cells were harvested, lysed by freeze–thaw cycling, and used for recombinant virus screening. The purity of recombinant viruses was achieved after 6 or 8 plaque-purification rounds for rSWPV-VP60 or rSWPV-VP60-His, respectively. To verify that the VP60 and VP60-His genes had been correctly inserted in rSWPV-VP60 or rSWPV-VP60-His, viral DNA was extracted from PK15 cells mock-infected or infected at 3 MOI for 72 h with the different viruses, as previously described, and the correct insertion of both genes was confirmed by PCR analysis using primers TK309-F and TK309-R (Table 1), annealing in the SWPV TK gene-flanking regions.

### Immunofluorescence assay analysis

The recombinant RHDV VP60 protein expression was identified by IFA (Rohde et al., 2011). PK15 cells in 96-well plates were infected with rSWPV-VP60 or rSWPV-VP60-His at an MOI of 0.01 followed by incubation at 37°C and 5% CO_2_ for 72 h, cells were fixed with Pre-cooled methanol and acetone 1:1 mixture at 4°C 15 min. After 3 times of washing with cold PBS (5 min each), the cells were permeabilized in 0.05% Triton X-100 for 30 min at room temperature. The cells were subsequently blocked with 5% bovine serum albumin (BSA) in PBS for 1 h at 37°C and then incubated with the mouse monoclonal antibody 4D5 directed against RHDV VP60 protein for 2 h at 37°C. After washing five times with PBS containing 0.05% Tween 20 (PBS-T), the cells were incubated for 1 h at 37°C with RBITC-conjugated goat anti-mouse secondary antibody (1:500 dilution; Solarbio, China). We used DAPI (4′,6′-diamidino-2-phenylindole; Solarbio, China) to stain the cell nuclei. After being washed three times with cold PBS-T, the culture plate was observed under a fluorescence microscope at 100× (Nikon Eclipse Ts2R, Japan).

### SWPV replication kinetics

PK15 cell monolayers grown in 6-well plates were infected with SWPVs at 5 MOI. After adsorption at 37°C for 4 h, DMEM containing 5% FBS was added, and the plates were incubated at 37°C with 5% CO_2_. Cell supernatants were harvested in triplicates at 0, 24, 48, 72, 96, and 120 h post-infection (hpi), by Freeze–thaw three times, and used for virus titration using the real-time PCR. (The primers and probe sequences are shown in Table 1). Virus titration at each point was carried out in three replicates.

### SDS-PAGE and western blotting analysis

Recombinant SWPVs were used to infect PK15 cells at an MOI of 5, the supernatant was replaced with serum-free DMEM for maintenance 48 h after infection, and cell cultures were harvested 4–5 days after infection (all cells were dead and shed). Harvested cells and supernatant were separated by centrifugation at 12,000 rpm for 10 min at room temperature in a microcentrifuge. Both the supernatant and the cell lysates were subjected to 10% SDS-PAGE and transferred onto polyvinylidene fluoride (PVDF) membranes. VP60 and VP60-His were probed with anti-VP60 mouse monoclonal antibody 4D5. Furthermore, PK15 cells were seeded in 6-well plates and infected at 5 MOI with rSWPV-VP60 or rSWPV-VP60-His to test the VP60 expression level at different time points post-infection. Culture fluids and cells were collected from 6-well plates, in volumes of 2.5 ml, and lysed by freeze–thaw three times. Next, the lysates were clarified by centrifugation at 12,000 rpm for 10 min to remove the cell lysates. Then, the supernatants were analyzed by western blot analysis. HRP-conjugated anti-mouse antibodies (TransGen, China; diluted 1:2,000) were used as the secondary antibodies. The immunocomplexes were detected with an HRP-luminol enhanced-chemiluminescence system (ECL Plus; Bio-Rad, United States).

### Transmission electron microscopy detection

To verify whether VP60 proteins self-assemble into VLPs, PK15 cells in T75 flasks were infected with recombinant SWPVs with an MOI of 5, serum-containing DMEM was replaced with serum-free DMEM at the 48 hpi, and the culture was continued up to 120 hpi. After three freeze–thaw cycles, the suspension was centrifuged at 12,000 rpm for 10 min at 4°C. Supernatants of infected PK15 cell cultures were filtered and concentrated 10-fold using Amicon Ultra 100 K centrifugation columns, negatively stained with 1% uranyl acetate (pH 4.5), and analyzed in a TEM (H-7500, Hitachi, Japan) with an operating voltage of 80 kV (Guo et al., 2016).

### Vaccination and virus challenge

To prepare immunogens for vaccination trials, the SWPVs were inoculation into PK15 cells cultured in serum-free DMEM. The suspended solution underwent three freeze–thaw cycles and was centrifuged at 1,000 × *g* for 10 min to remove cell debris. The virus titer of the harvested virus suspension was measured and diluted uniformly to 10^6^ PFUs/ml. The virus suspension was then dissolved in 0.05% β-propiolactone at 4°C for 24 h to inactivate the SWPVs, and then hydrolyze the β-propiolactone in a water bath at 37°C for 2 h. Inactivation was confirmed by PK15 cells. After inactivating the SWPV, the antigen and aluminum vaccine adjuvants (General Chemical Corp, United States) were mixed in a 9:1 ratio under agitation.

For immunization, 24 2-month-old specific-pathogen-free New Zealand white rabbits that lacked anti-RHDV antibodies were used and raised in isolated cages. The rabbits were randomly divided into four groups (6 per group) and vaccines were administered once intramuscularly with 1 ml of (1) commercial RHDV-inactive vaccine (Nanjing Tianbang, China), (2) inactivated SWPV-JX20G, (3) inactivated rSWPV-VP60, and (4) inactivated rSWPV-VP60-His. Blood samples were taken on weeks 0, 1, 2, 3, 4, 5, 6, 7, 9, 11, and 13 post-intramuscular immunization, and serum was prepared to determine RHDV virus-specific IgG by ELISA or hemagglutination inhibition (HI) assay. After 13 weeks of initial inoculation, all rabbits were challenged with classic RHDV infected rabbit liver tissue homogenated by inoculated intramuscularly of 2^10^ hemagglutination units. Rabbits were then observed daily for clinical signs and monitored for 10 days after the challenge Rabbits that were alive at the end of the study period were euthanized. Immunohistochemical (IHC) analysis was used to identify lesions and antigenic distribution in various tissues. All the immunization trials involving rabbits were approved and performed in compliance with the guidelines of the Animal Research Ethics Board of Jiangxi Agricultural University, CAAS (no. JXAULL-2021-15).

### Enzyme-linked immunosorbent assays

RHDV-specific antibodies in immunized rabbits were detected by indirect ELISA assays. To prepare the encapsulated antigen, PK15 cells were infected with rSWPV-VP60-His, maintained in serum-free DMEM for 5 days, and the freeze–thaw supernatant was collected to purify the VP60-His protein according to the steps of the His-tagged protein purification kit (CWBIO, China). The purified recombinant VP60-his protein was used to coat 96-well plates at a concentration of 5 μg/ml in a volume of 100 μl per well. Test sera were titrated in duplicate using 1,000-fold dilutions. The plates were incubated at room temperature for 1 h, then washed and incubated for 1 h with detection antibody, goat anti-mouse IgG-horseradish peroxidase (HRP; 1:2000 dilution; TransGen, China). The plates were washed and incubated using 3,3′,5,5′-tetramethylbenzidine (TMB) at room temperature in the dark; the reaction was stopped after 15 min using 1 M H_2_SO_4_ and read out at 450 nm (Tecan Sunrise ELISA plate reader). The sera from the rabbit inoculated with inactivated SWPV-JX20G were used as the negative control the results are expressed as OD_450_ nm values.

### Hemagglutination inhibition assay

We performed a hemagglutination inhibition assay to determine the RHDV HI antibody titer in the serum. The antigen used in this study was prepared using infected rabbit liver collected freshly at death as described previously (Berninger and House, 1995). Sera were inactivated at 56°C for 30 min and then subjected to a second serial dilution in a 96-well V-bottom titration plate containing 25 μl PBS. RHDV antigen (25 μl, 8 HA units) was added except for the last row which served as a control. Serum dilutions were 1:2 to 1:2^14^. Plates were incubated at 25°C for 1 h. A volume of 25 μl of 1% human O-erythrocyte suspension was added to each well and incubated for another 30 min. Additional positive sera, negative sera, erythrocytes, and antigen were used as controls. The highest serum dilution leading to complete inhibition was considered as the endpoint. The geometric mean titer was expressed as the log2 value of the reciprocal of the highest dilution indicating HI (Song et al., 2017).

### Immunohistochemistry

Surviving rabbits were executed on day 10 after the challenge. Lung, liver, and spleen tissues were collected from rabbits that died of infection or were euthanized at necropsy and were evaluated histopathologically (Prieto et al., 2000). Tissues were fixed, dehydrated, paraffin-embedded, sectioned, and immunohistochemically examined using mouse monoclonal primary antibody 4D5 and horseradish peroxidase (HRP)-conjugated goat anti-mouse secondary antibody (ZSGB-Bio, China).

### Statistical analysis

Data for antibody response and HI titer were compared between the three groups with one-way ANOVA using GraphPad Prism version 9 (GraphPad Software, San Diego, CA, United States). *p* < 0.05 was considered to indicate a significant difference.

## Results

### Generation and *in vitro* characterization of the recombinant swinepox virus

**Figure 1:**
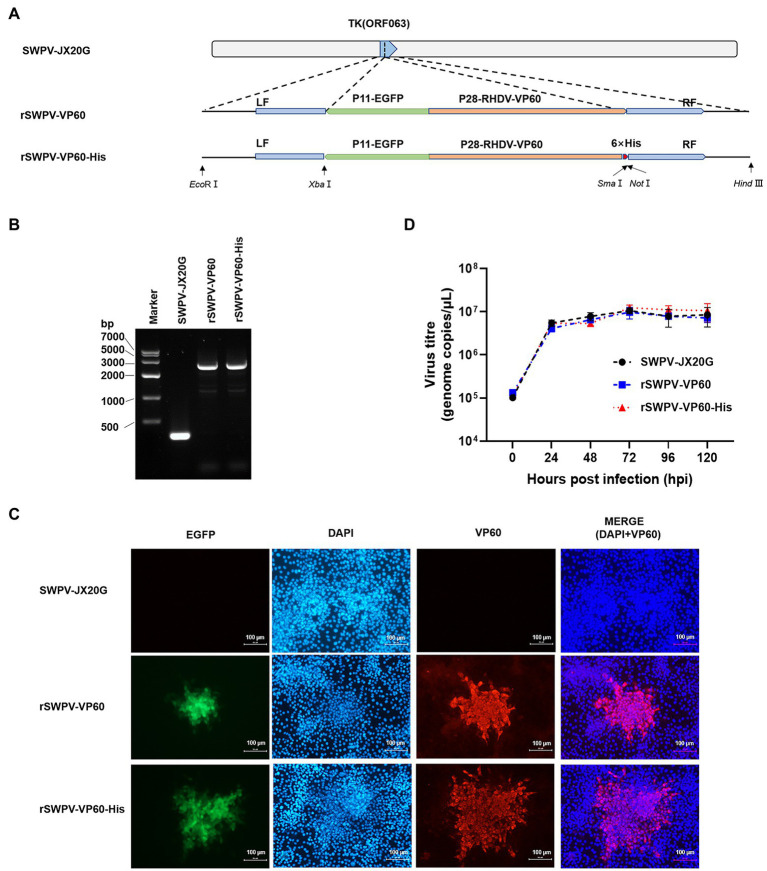
Construction and characterization of recombinant strains in PK15 cells. **(A)** Diagram indicating recombinant swinepox virus constructs.TK, thymidine kinase gene; LF, left flank; RF, right flank; EGFP, enhanced green fluorescent protein; P11, vaccinia virus P11 promoter; P28, vaccinia virus P28 promoter; RHDV VP60, rabbit hemorrhagic disease virus VP60 protein; His, Histidine. **(B)** PCR analysis of the recombinant virus. **(C)** Immunofluorescence assay detection of the recombinant swinepox virus. The fluorescence of EGFP was observed by confocal microscopy directly, identification of VP60 by IFA assay using an anti-VP60 antibody, respectively. Red and green fluorescence was observed in strain rSWPV-VP60 and rSWPV-VP60-His, but not in strain SWPV-JX20G. The scale bar represents 100 μm. **(D)** Single-step growth curve. Infected cells were harvested at the indicated hours post-infection. The virus titer was determined by qPCR. Wild-type SWPV JX20G was used as controls. Titers (genome copies/μl) represent the averages of the results of three independent experiments. The titer of the recombinant swinepox virus was highest at 72 hpi.

To develop novel RHDV vaccines with new antigen production modalities, we developed two rSWPV vaccine candidates expressing the RHDV VP60 structural gene, a VP60 protein identical to the natural amino acid sequence, and a purifiable VP60-His protein with a six histidine His tag added at the carboxyl terminus. Both candidates were generated by inserting a fluorescence marker and the target expression gene into the thymidine kinase (TK) locus of the wild virulent strain SWPV-JX20G (Figure 1A). PCR analysis of the correct generation and purity of the rSWPV-VP60 and rSWPV-VP60-His constructs using oligonucleotide annealing of the flanking region of SWPV TK confirmed the correct insertion of the VP60 gene without the presence of SWPV-JX20G (Figure 1B), and DNA sequencing of the amplified fragment confirmed the fidelity of the target gene sequence. Individual patches of PK-15 cells infected with SWPV-JX20G, rSWPV-VP60, and rSWPV-VP60-His were observed by fluorescence microscopy. All recombinant strains showed green fluorescence. IFA confirmed the expression of VP60 protein and showed by comparison with DAPI staining that the expressed protein was predominantly present in the cytoplasm, which is consistent with the replicative activity of cytoplasmic interstitial poxvirus (Figure 1C). Growth kinetic analysis in PK15 cells showed that rSWPV-VP60 and rSWPV-VP60-His have similar viral growth to their SWPV-JX20G parental viruses. Thus, constitutive expression of the VP60 protein and TK deletion does not affect the replication of the SWPV vector under permissive conditions (Figure 1D).

### Soluble expression of VP60 protein self-assembled into VLPs

**Figure 2:**
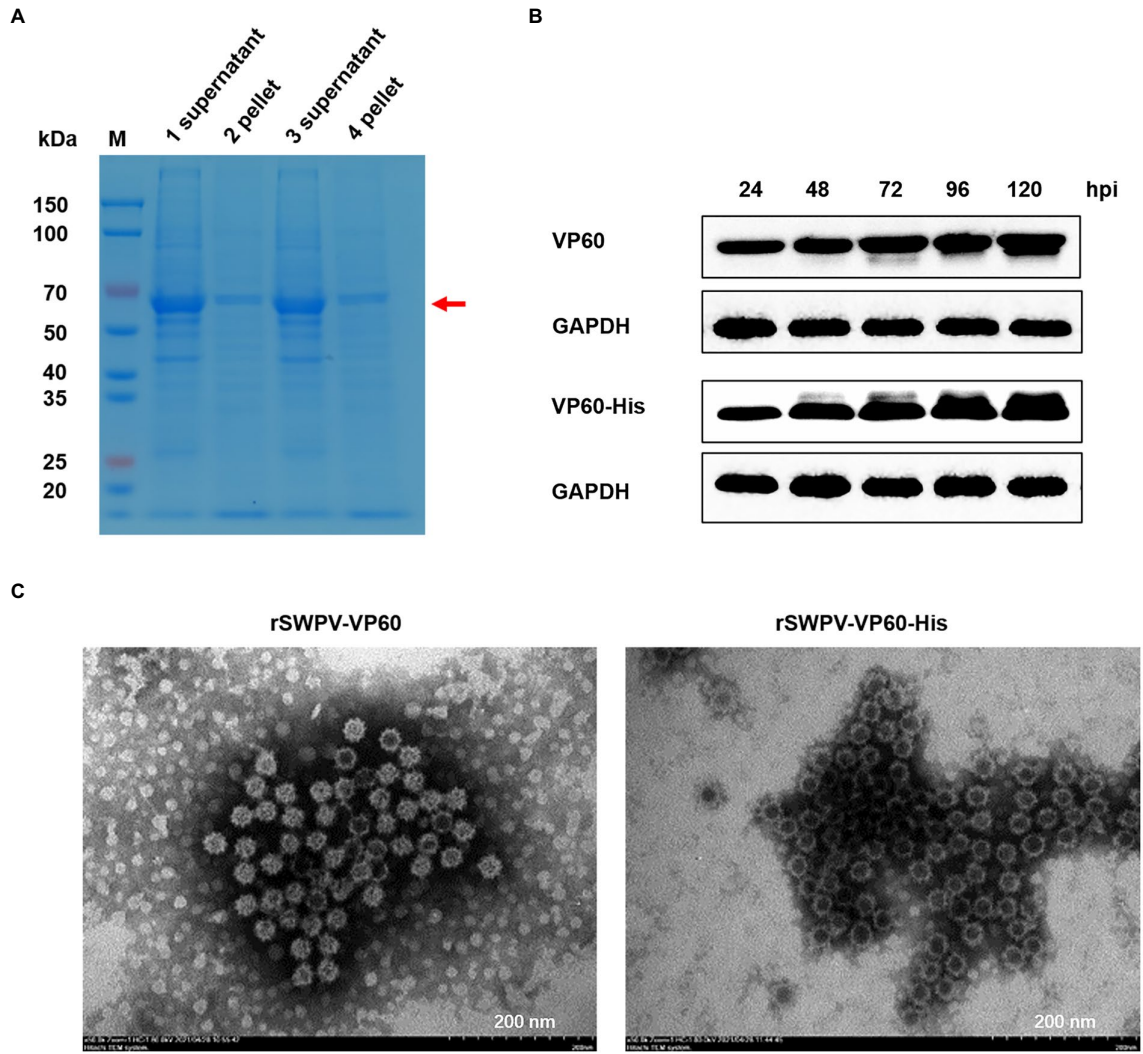
Analysis of recombinant protein expression. **(A)** Expression pattern of the recombinant protein as studied by SDS-PAGE. Samples from freeze–thaw supernatant and pellet (lanes 1 and 2, rSWPV-VP60; lanes 3 and 4, rSWPV-VP60-His), respectively, were run in SDS-PAGE gels under reducing conditions. **(B)** To determine the kinetics of protein expression, 100% confluent PK15 cells were infected with rSWPV-VP60 or rSWPV-VP60-His at an MOI of 5. Equal amounts of total freeze–thaw supernatant, harvested at the indicated time points post-infection, were analyzed by SDS-PAGE, followed by western blotting using the antibodies described in Materials and Methods. A representative blot of the GAPDH as a control protein is also shown. The expression level of VP60 proteins peaked at 120 hpi. **(C)** TEM images of RHDV VP60 VLPs. Supernatants of infected PK15 cell lysates were filtered and concentrated 10-fold using Amicon Ultra 100 K centrifugation columns and dialyzed in PBS buffer. Samples were negatively stained with 0.5% aqueous uranyl acetate. TEM analysis showed that VP60 proteins assembled into VLPs with diameters of 33–40 nm. Bar = 200 nm.

To determine the form of VP60 protein expression, SDS-PAGE was performed on the lysate supernatant and precipitate of infected PK15, respectively. The results showed that there was a 60 kDa specific target protein band in the supernatant of the lysate, and both VP60 proteins existed in soluble form (Figure 2A). Western blotting showed protein expression in PK15 cells infected by the rSWPV vaccine candidates, with VP60 protein expression levels increasing with time post-infection, with the highest expression at 120 h post-infection (complete lesion shedding of cells; Figure 2B). To identify whether the expressed VP60 protein self-assembles to form VLPs, PK15 cells infected with rSWPV-VP60 or rSWPV-VP60-His of freeze–thaw supernatant were filtered and concentrated 10-fold using Amicon Ultra 100 K centrifugation columns and dialyzed in PBS buffer. Samples were negatively stained with 0.5% aqueous uranyl acetate and visualized by electron microscopy analysis. TEM showed a high concentration of spherical particles with diameters ranging from 33–40 nm (Figure 2C). This demonstrates the successful assembly of RHDV VLPs in PK15 cells.

### Humoral immune responses in the rabbit model

**Figure 3:**
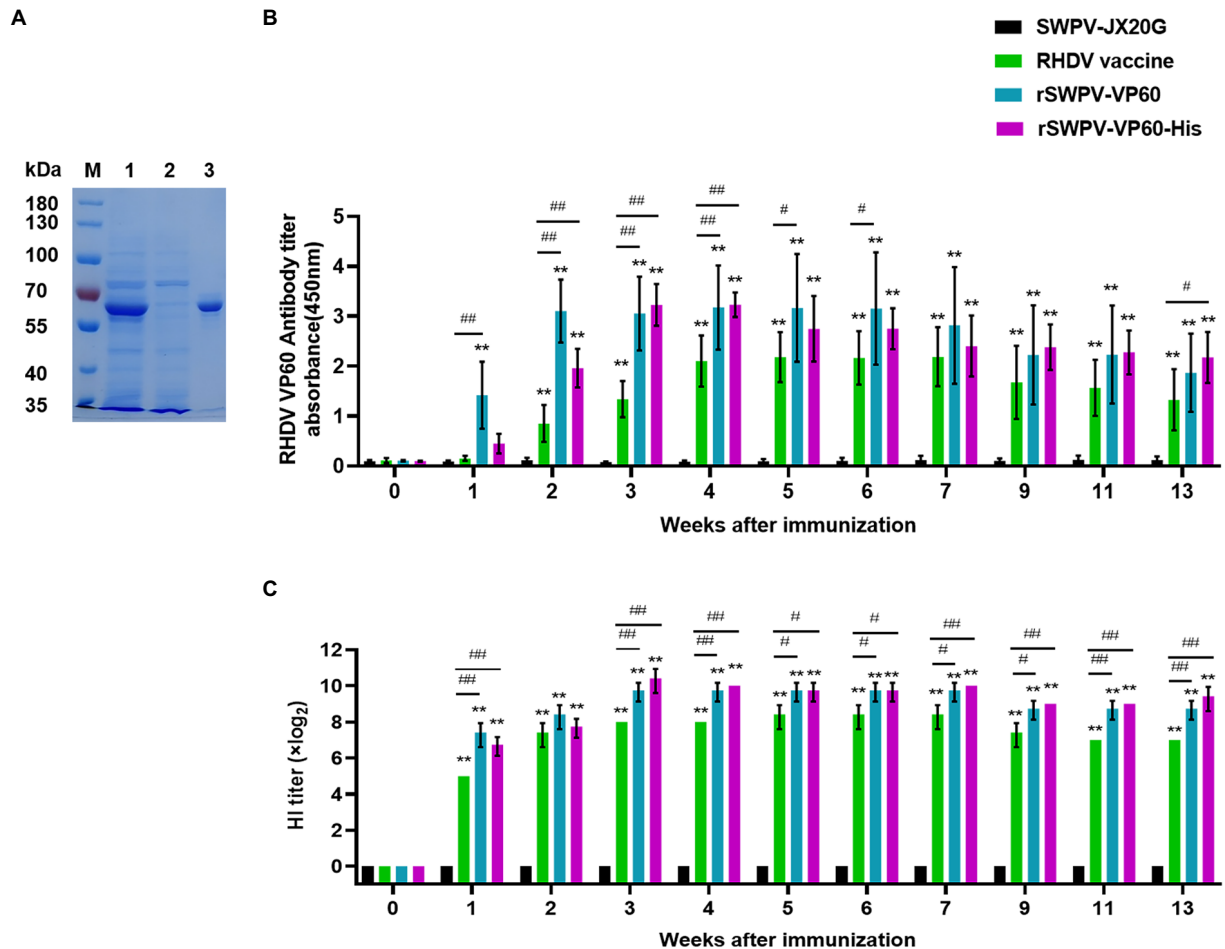
Humoral immune response to the commercial RHDV vaccine, rSWPV-VP60 or rSWPV-VP60-His in rabbits. **(A)** Purified VP60-His protein as capture antigen for ELISA. Lane 1: VP60-His protein expressed using the swinepox virus/PK15 system; land 2: the effluent of protein purification; lane 3: the purified VP60-His protein. **(B)** Anti-VP60 antibody levels were measured by ELISA in serum samples of pre-immune or immunized rabbits using purified recombinant VP60-His. **(C)** The hemagglutination inhibition (HI) titers of serum samples from immunized rabbits were detected, *p < 0.05, **p < 0.01; #*p* < 0.05, ##*p* < 0.01, using one-way ANOVA.

Antibodies against RHDV-VP60 protein play a decisive role in controlling RHD progression. Therefore, to assess the ability of the rSWPV vaccine candidate to induce humoral immune responses against RHDV, we detected the presence of specific IgG in the sera of immunized rabbits by ELISA using VP60-His protein as the encapsulated antigen (Figure 3A). Specifically, the rSWPV-VP60, rSWPV-VP60-His, and commercial vaccine groups induced specific anti-VP60 antibodies, whereas those immunized with the SWPV-JX20G group did not. The antibody potency was higher in rabbits immunized with both rSWPVs than in those with the commercial vaccine. After 1 week of immunization, there was a significant difference in titers between rabbits immunized with the recombinant swinepox virus and those immunized with the commercial vaccine, with the rSWPV-VP60, rSWPV-VP60-His, and commercial vaccine groups reaching the highest levels at 2, 3, and 5 weeks after immunization, respectively (Figure 3B).

The HI assay was used to test the ability of each serum sample to neutralize the virus. All three immunization groups, except the control group immunized with SWPV, produced serum with the ability to neutralize the virus at 1 week after immunization. rSWPV-VP60, rSWPV-VP60-His, and the commercial vaccine group reached the highest level of ability to inhibit RHDV hemagglutination at 3, 3, and 5 weeks after immunization, respectively (Figure 3C).

### Immune protection against RHDV challenge

**Figure 4:**
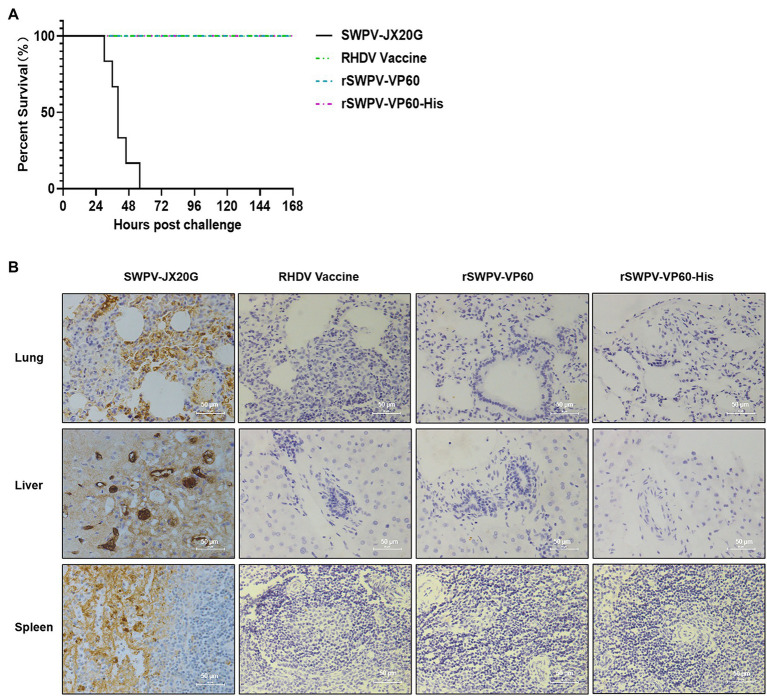
Protective efficacy of the rSWPVs in rabbits. **(A)** Survival curves of rabbits after a lethal challenge with the RHDV. All the rabbits in 4 groups (6 rabbits in each group) were challenged with RHDV at 13 weeks after immunization and survival rates were recorded for 7 days after the challenge. **(B)** IHC analysis of lung, liver, and spleen from immunized rabbits. Images of IHC analysis presented that classic RHDV VP60 was predominantly located in the cellular cytoplasm of rabbits immunized with the DMEM, but not in that of rabbits immunized with RHDV Vaccine, rSWPV-VP60, and rSWPV-VP60-His. Magnification ×400, Bar = 50 μm.

To examine the immunoprotective efficacy, all experimental rabbits were challenged intramuscularly with 1 ml RHDV (1,024 HA units) at week 13 after the initial immunization. The results showed that all rabbits in the SWPV-JX20G strain group died within 48 h of immunization and that rabbits immunized with rSWPV-VP60, rSWPV-VP60-His, or commercial vaccine were completely protected from RHDV challenge (Figure 4A).

The lung, liver, and spleen of rabbits were taken for histopathological evaluation and pathogen distribution assay (immunohistochemical assay using anti-VP60 monoclonal antibody). SWPV-JX20G-treated group showed significant tissue damage in the lung, liver, and spleen with massive distribution of RHDV antigen. rSWPV-VP60-and rSWPV-VP60-His-immunized group and commercial vaccine immunized groups did not show significant histopathology and antigenic staining (Figure 4B).

## Discussion

Rabbit hemorrhagic disease (RHD) is a highly contagious and fatal viral infection that can cause severe economic losses to both wild and domestic rabbits. The development of effective recombinant subunit vaccines is critical, especially for pathogens such as RHDV that are difficult to proliferate *in vitro*. Although Zhu has successfully bypassed this obstacle by constructing mutant RHDV (mRHDV) using reverse genetic techniques (Zhu et al., 2017), the viral titers are low, and efficacy and safety remain to be proven. Commercially available vaccines include RHDV-VLPs produced from conventional inactivated viruses and baculovirus-insect cell expression systems. However, the cost of producing RHDV-VLPs from insect cells is quite high (Perez-Filgueira et al., 2007), which is important when producing at scale, so it is necessary to develop alternative protein expression systems.

Recombinant swinepox virus systems have been successfully developed as vectors for the delivery of multiple vaccine antigens (Yuan et al., 2018). Suitable insertion sites for heterologous genes have been described, and the ability of SWPV-based recombinants to induce strong immune responses, including mucosal immunity, has been validated (Lin et al., 2017; Yuan et al., 2017). In this study, we constructed an rSWPV genome carrying a VP60 expression cassette and generated recombinant swinepox virus by transfecting PK15 cells with the recombinant genome. The viral titers and proliferation curves of rSWPV were similar to those of SWPV, indicating that the deletion of SWPV TK did not affect viral replication, consistent with previous experiments. As demonstrated by IFA and western blotting analysis, cells infected with rSWPV correctly express the RHDV VP60 protein. It is controlled by the vaccinia virus P28 promoter as expected (Fang et al., 2016).

VLPs resemble a true virus in structure and result from the self-assembly of viral structural proteins. Due to the lack of viral genetic material within the capsid, VLP is non-infectious and has a safety profile similar to that of subunit vaccines (Dalton et al., 2021). However, in contrast to subunits, VLPs exhibit their antigenic epitopes in a structured, repetitive manner that can serve as a pathogen-associated molecular model (PAMP). In this way, co-stimulatory signals can be used by antigen-presenting cells to activate T-lymphocytes with high immunogenicity (Miao et al., 2019). Soluble expression of structural viral proteins has been reported to be necessary for VLP self-assembly, while VP60 can form VLPs in a variety of expression systems (Nagesha et al., 1995). SDS-PAGE results showed that VP60 protein was expressed in a soluble form in the recombinant swinepox system in this study. Electron microscopy results showed that the cap protein VP60 produced in PK15 formed VLPs similar in size and morphology to the natural RHDV particles, with cup-shaped structures on the surface (Barcena et al., 2015).

SWPV vectors are known to induce strong mucosal and systemic immune responses against antigens encoded by exogenous insertion genes. We then formulated VLPs vaccines by mixing inactivated recombinant viral cultures (10^6^ PFUs/ml) with aluminum gel adjuvant and immunized rabbits with it. Humoral immunity plays an important role in the fight against RHDV infection (Ferreira et al., 2008). Our results showed that highly specific antibody titers against RHDV were strongly induced in rabbits detectable at 7 days after vaccination, peaked after 4 weeks, and were maintained during the ongoing monitoring for the following 13 weeks. In addition, studies have shown that a HI titer of 1:2^4^ or more was sufficient to protect rabbits from lethal RHDV challenge (Fernandez et al., 2013; Yang et al., 2015; Zheng et al., 2016), and our results showed that all immunized groups had HI titers higher than 1:2^4^ at 7 days post-immunization. All animals survived above the lethal dose of challenge virus for field infection without any clinical signs, confirming the results. Rabbits challenge with RHDV often develop acute necrotizing hepatitis and disseminated intravascular coagulation in the lung, liver, spleen, and other solid organs (Abrantes et al., 2012). IHC analysis revealed local histopathological changes in the lungs, liver, and spleen of the control group after the attack with RHDV, which were characterized by antigenic staining. However, these changes were not observed in the organs of rabbits immunized with recombinant SWPV or Commercially vaccines. This finding suggests that immunization of recombinant swinepox virus could fully protect against lethal RHDV challenge in rabbits.

Collectively, this study adds another example of the successful use of this new vector virus. It demonstrates that recombinant SWPV can be used as a vector for RHDV antigen expression. Based on the fact that RHDV type 2 has been reported in China and the poxvirus has high exogenous gene capacity, this vector has the potential to be used for developing a bivalent vaccine candidate strain.

## Data availability statement

The original contributions presented in the study are included in the article/supplementary material, further inquiries can be directed to the corresponding author.

## Ethics statement

The animal study was reviewed and approved by Jiangxi Agricultural University Animal Experiment Ethics Review Committee.

## Author contributions

CL: conceptualization, methodology, formal analysis, data curation, visualization, and writing—original draft. SD: methodology, data curation, validation, writing—review and editing, project administration, supervision, resources, and conceptualization. HH: validation and formal analysis. ML and XH: validation and visualization. XLiu and YB: data curation, validation, and formal analysis. XLi and WY: resources and validation. FL: funding acquisition, project administration, resources, and supervision. All authors contributed to the article and approved the submitted version.

## Funding

This work was supported by grants from the National Natural Science Foundation of China (Grant No. 31460666). Part of this work was also supported by the Technology System of Modern Agricultural Swine Industry of Jiangxi Province (JXARS-03).

## Conflict of interest

FL was employed by Jiangxi Jinyibo Biotechnology Company.

The remaining authors declare that the research was conducted in the absence of any commercial or financial relationships that could be construed as a potential conflict of interest.

## Publisher’s note

All claims expressed in this article are solely those of the authors and do not necessarily represent those of their affiliated organizations, or those of the publisher, the editors and the reviewers. Any product that may be evaluated in this article, or claim that may be made by its manufacturer, is not guaranteed or endorsed by the publisher.
